# Neurotrophic keratopathy: current challenges and future prospects

**DOI:** 10.1080/07853890.2022.2045035

**Published:** 2022-03-04

**Authors:** Erin NaPier, Matthew Camacho, Timothy F. McDevitt, Adam R. Sweeney

**Affiliations:** a John A. Burns School of Medicine, University of Hawaii, Honolulu, HI, USA; b Section of Ophthalmology, Department of Surgery, John A. Burns School of Medicine, University of Hawai’i, Honolulu, HI, USA

**Keywords:** Neurotrophic keratitis, neurotrophic keratopathy, corneal anaesthesia, corneal nerves, corneal ulcer, Mackie classification, autologous serum drops, neurotization, novel therapies

## Abstract

Neurotrophic keratopathy (NK), or neurotrophic keratitis, is a degenerative condition that results from decreased innervation to the cornea. The cornea is innervated by the ophthalmic branch of the trigeminal nerve. Neurotrophic keratopathy is most commonly caused by herpes keratitis however, any condition that disrupts the normal corneal innervation can cause NK. Neurotrophic keratopathy is a clinical diagnosis and is classified into three stages based on the disease severity. Stage 1 has mild epithelial defects, such as punctate keratopathy, stage 2 disease has persistent epithelial defects, and stage 3 is defined by the presence of ulcers. Current treatment modalities consist of medical and surgical options. Stage 1 is treated with lubrication through artificial tears, eyelid taping, and punctal plug/cautery. Stage 2 treatment can involve therapeutic contact lenses, topical autologous or allogenic serum, tarsorrhaphy, botulinum toxin injections, and possibly anti-inflammatory medications. Stage 3 disease may require human nerve growth factor, amniotic membrane transplantation, conjunctival flap, or corneal neurotization. New therapies, such as matrix regenerating therapy, plasma rich in growth factors, Thymosin β4, Substance P/Insulin like growth factor-1, and nicergoline represent exciting future options.

**KEY MESSAGES**
- Neurotrophic keratopathy is a rare degenerative disease defined by decreased innervation to the cornea that is associated with significant morbidity.
- Treatment options range from lubrication alone to various medical and surgical treatments.
- Matrix regenerating therapy, plasma rich in growth factors, Thymosin β4, Substance P/Insulin like growth factor-1, and nicergoline are exciting novel therapies that will influence how neurotrophic keratopathy is treated in the future.

## Introduction

Neurotrophic keratopathy (NK) is a rare, degenerative condition characterized by decreased innervation of the cornea that affects <1.6 per 10,000 people [1,2]. While there is an extensive list of possible causes, prior corneal herpetic infection is the most common aetiology [1]. Notoriously difficult to manage, NK may lead to significant patient morbidity or decreased quality of life. Symptoms are primarily based on stage and severity, ranging from asymptomatic to varying degrees of impaired vision [2]. Appropriate medical and surgical treatment options are based on the severity of the disease. Treatment options range from lubrication alone to multimodality management including aggressive topical and surgical treatments. Recent advances in therapy include strategies aimed at re-innervating the cornea including topical nerve growth factors (NGF) and corneal neurotization. Several novel therapies, such as matrix regenerating therapy, plasma rich in growth factors (PRIGF), Thymosin β4, Substance P/Insulin-like growth factor-1 (IGF-1), and nicergoline are under investigation. The purpose of this article is to provide a review of current literature on neurotrophic keratopathy with a focus on current and novel treatments.

## Pathogenesis

The cornea is an avascular, prolate-shaped connective tissue that provides refractive power, structural integrity, and antimicrobial functions in the anterior portion of the eye [3]. It is mainly innervated by the nasociliary nerve of the ophthalmic branch (V1) of the trigeminal nerve and occasionally by sensory nerves of the maxillary branch (V2) innervate the inferior cornea [3,4]. Additionally, there is autonomic innervation from the superior cervical ganglion (sympathetic) and ciliary ganglion (parasympathetic), however, the role and density of these nerves remain unclear [4]. It is estimated that the subbasal plexus of the cornea has ∼19,000–44,000 axons [4]. Corneal innervation is critical in providing trophic factors (e.g. substance P, calcitonin gene-related peptide, acetylcholine, serotonin, neuropeptide Y) which help to maintain a healthy corneal epithelium. The mutual relationship between corneal nerves and epithelium is essential for corneal homeostasis as epithelial cells secrete neurotrophic factors (e.g. nerve growth factor and ciliary neurotrophic growth factor) with parallel effects on corneal nerves. Loss of innervation results in decreased metabolism and mitosis of epithelial cells, consequently leading to epithelial breakdown and hindered corneal healing [1,5,6]. This may result in persistent epithelial defects, ulceration, stromal melting, and perforation [7].

## Aetiologies

The numerous causes of NK share the common pathway of disrupting corneal innervation. Herpes simplex virus (HSV) and herpes zoster virus (HZV) infections, are the most common causes of NK and account for ∼27–32% of NK cases [6–8]. Other common causes include chemical burns, extended use of contact lenses, and iatrogenic causes, such as corneal surgeries or neurosurgical injury to the trigeminal nerve. Saad et al. reported 34.2% of patients with multifactorial causes of NK and 8.2% with idiopathic aetiologies of NK [7]. A comprehensive list of causes is provided in Table 1 [1,6,11].

**Table 1.** Causes of neurotrophic keratitis.

Infections	HSV, HZV, Mycobacterium leprae
Ocular surface disease	Chronic blepharitis, Chemical burns, contact lens use, entropion, lattice and granular dystrophy, ocular trauma
Iatrogenic	Corneal surgery (laser-assisted *in situ* keratomileusis, corneal incisions, keratoplasty), vitrectomy, multiple ocular surgeries, corneal grafting, refractive surgery
Medications	Anaesthetics, timolol, betaxolol, trifluridine, sulfacetamide, diclofenac sodium topical anaesthetics (timolol, betaxolol [9], sulfacetamide, diclofenac [10])
CN V palsy	Neoplasm, aneurysm, facial trauma, surgery for trigeminal neuralgia
Systemic conditions	Diabetes, vitamin A deficiency, multiple sclerosis
Congenital/genetic	Riley day syndrome (family dysautonomia), moebius corneal hypoesthesia, goldenhar-gorlin syndrome, and congenital corneal anaesthesia [6]
Other	Long term antipsychotic or antihistamine use, Adies syndrome, increasing age

HSV: Herpes simplex virus; HZV: Herpes zoster virus.

## Epidemiology

Neurotrophic keratopathy is classified as an orphan disease (ORPHA137596). Extensive epidemiological data is not available. Previous literature derived from epidemiological data on conditions associated with NK suggested that 1–5 per 10,000 people are affected [2,12–14]. A recent, retrospective, observational monocenter study at the Fondation Ophtalmologique Adolphe de Rothschild Hospital (Paris, France) screened over 300,000 patients and reported a prevalence of 11 per 10,000 patients [7]. Saad et al. concluded that previous studies incorporated only some of the aetiologies of NK suggesting the real frequency is much higher than once thought [7].

## Clinical presentation

Close monitoring of NK is imperative as its progression is often asymptomatic. There is often a discrepancy between ocular signs and symptoms due to corneal anaesthesia.

The most common presenting symptoms of NK include redness, sensitivity to light, dry eye, reduced visual acuity, blurred vision, and eye fatigue [15]. The most frequently reported patient complaints to include driving impairment, reading impairment, difficulty watching television, and concern with potentially losing their eyesight due to NK [15]. Patients with advanced disease may present with late signs, such as severe vision loss due to corneal scarring or even with corneal perforation.

## Classification

The Mackie classification scheme identifies neurotrophic keratopathy fitting into one of 3 stages [1,16]. Stage 1 is characterized by epithelial alterations, such as punctate keratopathy, hyperplasia, irregularity, and superficial neovascularization. Rose Bengal staining of the conjunctiva can show epithelial compromise. Stage 2 has persistent epithelial defects (PED) that are commonly found in the superior half of the cornea. Surrounding epithelial edoema can easily detach and enlarge the defect. The edges of the PED can appear smooth with rolled edges. Stage 2 can also present with folds in Descemet's membrane and an inflammatory reaction in the anterior chamber. Stage 3 is characterized by corneal ulcers that may progress to perforations or stromal melting.

## Diagnosis

Neurotrophic keratopathy is a clinical diagnosis. A diagnosis of NK is suspected based on the patient’s history and is confirmed by physical exam findings. The exam should include a thorough cranial nerve exam. Assessing sensation in V1, V2, and V3 distribution can localize nerve involvement. Eyelids should be inspected with a special focus on position and movement to determine if exposure keratopathy, involutionary changes, or blepharitis may be exacerbating the disease. Blink rate can be decreased in bilateral NK, which can predispose to exposure keratopathy. The unilateral disease usually does not affect the blink rate [2].

Common corneal findings include central corneal disease characterized by PED, corneal thinning, neovascularization, ulcer with rolled edges, corneal melt, and opacification from scarring with progression over time [17]. In the presence of a corneal ulcer, cultures should be obtained. The intact epithelium is integral for preventing infections and thus fluorescein staining should be utilized during the exam. Infectious signs, such as a fluffy infiltrate, mucus development, or significant palpebral conjunctival reaction are not routinely present. Infectious aetiologies, however, must be ruled out before a diagnosis of NK can be made.

It is critical to measure corneal sensitivity before the instillation of examination drops to determine the presence of NK. Corneal sensitivity can be assessed qualitatively and quantitatively. Qualitative assessment can be performed using a thinned cotton whisp on a cotton swab. If the cornea is properly innervated, the patient will blink when cotton whisp touches the cornea. If innervation is compromised, the blink reflex may be diminished. Quantitative measurement of corneal sensitivity is commonly measured using a Cochet-Bonnet esthesiometer, which uses a nylon filament of adjustable length to test mechanical sensitivity (<5 mm suggests hyposensitivity, 6 mm suggests full sensitivity). A non-contact Belmonte esthesiometer can also provide quantitative data on mechanical, chemical, and thermal corneal sensitivity by using air puffs at varying temperatures, pressure, or CO_2_ concentrations.

Beyond corneal sensitivity testing, several other tests are helpful to identify comorbid conditions, classify the severity of NK, and monitor response to treatment or disease progression. Assessing tear film health is performed using the Schirmer test and tear osmolarity testing. Tear film health may be followed to assist in tracking subclinical disease progress [2,18]. *In vivo* confocal microscopy evaluates corneal nerve density in the stromal and sub-basal nerves. Eyes affected with NK can show decreased nerve density, decreased corneal epithelial and endothelial cell density, and increased hyperreflective keratinocytes. Patients who have had long disease duration typically have lower endothelial cell density. Unfortunately, cornea nerve evaluations involving confocal microscopy may have low reproducibility [2]. Ocular surface impression cytology identifies corneal conjunctivalization which can suggest limbal stem cell deficiency.

## Prognosis

The prognosis depends on the severity of the disease. In general, the more severe the lack of innervation to the cornea, the more likely NK is to progress [2]. A retrospective epidemiologic study showed that initial low corrected distance visual acuity, higher clinical severity, and older age negatively impacted the final corrected distance visual acuity (CDVA) [7]. Treatment should be initiated as soon as possible after diagnosis to prevent the progression of the disease.

## Treatment

**Figure 1.**
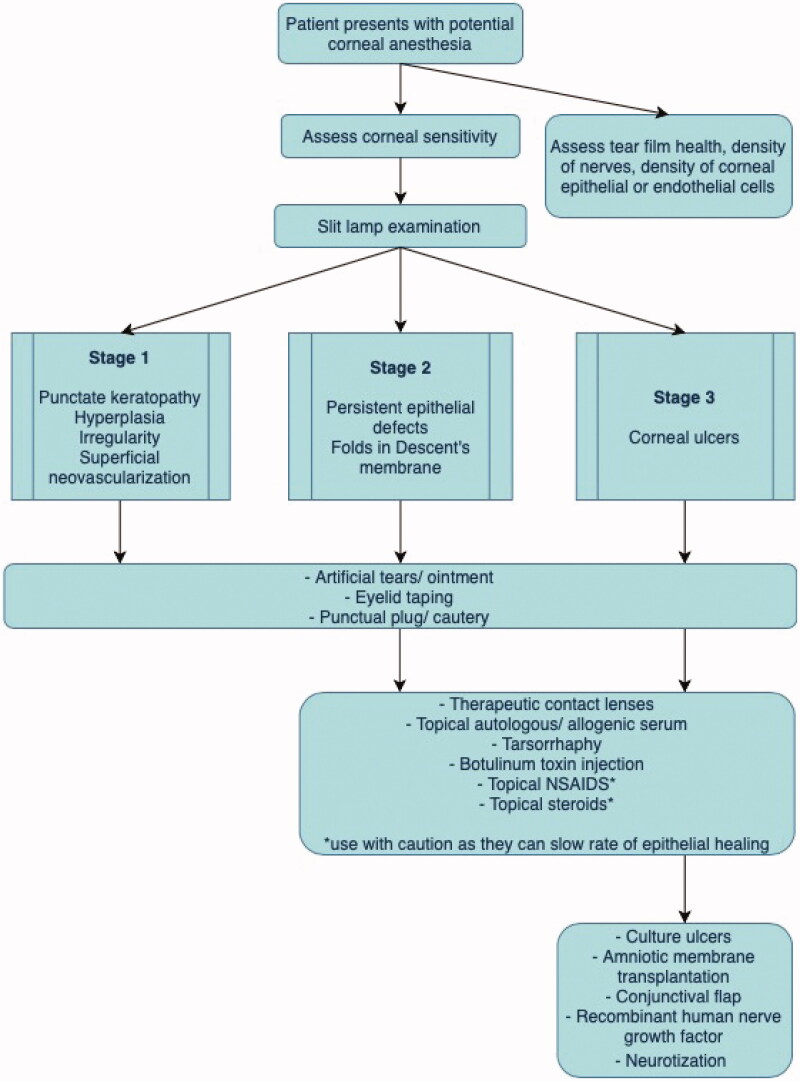
Overview of clinical management of neurotrophic keratitis according to the stage of the condition.

The goal of treatment is to prevent the progression of corneal damage and promote corneal healing. An overview of the clinical management and treatment options for NK is provided in Figure 1. Underlying conditions that are causing or contributing to NK should be addressed [2]. Collaboration with the cornea specialist and oculoplastic surgeon is helpful to provide patients with multimodal support. Eyelid dysfunction should be addressed concurrently with therapies directly treating the cornea to prevent further corneal damage. Treatments are tailored to the stage of disease except for artificial tears, which may be used at all stages of the disease [2]. As a patient’s disease progresses, treatment options associated with earlier stages should be continued.

Treatment options for stage 1 disease are primarily based on lubrication. Artificial tears, artificial tear ointment, eyelid taping, and punctal plugs may be prescribed individually or in combination [1,2,6,11]. Lubrication should be used on a scheduled basis and directed by the prescribing physician rather than an as-needed approach, as signs of inadequate treatment may not be evident to the patient.

If any aspect of inflammatory dry eye is evident, concurrent therapies with anti-inflammatory drops can be used with caution as it may inhibit the healing process [2]. Discontinuation of any offending topical medications may be utilized early after diagnosis to stabilize the cornea and simplify monitoring treatment [1,2,6,11].

For stage 2 disease, treatment goals involve promoting epithelial healing and preventing progression to corneal ulcers. Close monitoring is needed during stage 2 due to the propensity of rapid, asymptomatic progression.

Therapeutic contact lenses including a bandage contact lens or a scleral lens may be employed to protect the cornea and provide a reservoir of constant lubrication to the cornea [19]. Topical placement of autologous serum tears is often needed for recalcitrant disease. Autologous serum eye drops have similar osmolarity, pH, and composition to tears. Both contain growth factors and vitamins that maintain the epithelia and promote healing [6]. Autologous serum eye drops also contain IGF-1 which is not found in tears and has been shown to work synergistically with substance P to upregulate growth and migration of corneal epithelium [17,20]. Autologous serum eye drops are produced by sampling a patient's blood, removing cellular components *via* clotting and centrifuging the sample, then diluting it to a specific concentration [21]. A retrospective case series on 14 eyes of 11 patients showed complete healing of epithelial defects in an average of 17 days. Corneal sensitivities improved in 9 of 14 eyes (64.5%) and 5 eyes (35.7%) reached normal corneal sensitivity. The optimal concentration of autologous serum eye drops is unknown. Autologous serum drops are commonly 20% of the solution, however, 50% of serum solutions have also been successfully used [22].

The use of allogeneic serum, including serum from donor peripheral blood and umbilical cord blood rich in growth factors has shown promising results [23]. Allogeneic serum is used in patients with contraindications to autologous serum, such as inaccessible peripheral venous access and hematological pathology [24]. In a study by Yoon et al., application of 20% concentrated umbilical cord serum resulted in complete resolution of epithelial defects in 28 eyes (100%) and significant improvement in mean corneal sensitivity [25]. Healing of the epithelial defect occurred within four weeks in 22 eyes (78.6%). The authors report significantly higher levels of substance P and nerve growth factor in umbilical cord serum when compared to peripheral blood serum and tears. Allogeneic serum has been well-tolerated, though there is a risk for hypersensitivity and transmission of pathogens [26].

Surgical approaches for stage 2 disease include a temporary or permanent tarsorrhaphy [27]. The extent of the tarsorrhaphy may be dictated by the severity of the disease, the patient’s goals, and the status of the fellow eye though its utility is limited by poor cosmetic outcomes for patients [6]. An efficacious alternative is the injection of botulinum toxin into the levator muscle causing ptosis lasting 3-6 months [28]. Though similar in cosmetic appearance, this technique permits easier access and monitoring of corneal changes when compared to tarsorrhaphy [6].

Topical corticosteroids may be used judiciously as steroids may inhibit stromal healing and increase the risk of stromal melting and perforation [29]. Similarly, non-steroidal anti-inflammatory drugs (NSAIDS) should also be used with caution as they can also retard the rate of epithelial healing [1,2,30].

Stage 3 is an advanced form of NK and can be difficult to treat. Several medical and surgical approaches can be used. A new medical option for treatment is directed at replacing nerve growth factor (NGF) with topical instillation. On the surface of the eye, NGF is involved in the survival and healing of sensory and sympathetic neurons and immune responses [4,31]. A topical formulation of recombinant human NGF, cenegermin (Oxervate; Dompé Farmaceutici SpA, Milan, Italy), has become readily available in the USA in the last decade. A multi-center, randomized, double-masked trial demonstrated that patients treated with cenegermin topical drops had higher rates of corneal healing at 8 weeks in patients with NK and non-healing corneal defects compared to the vehicle control group [32]. Patients receiving cenegermin had a faster rate of corneal healing and slower disease progression. Additionally, cenegermin patients had a lower rate of disease recurrence in the 48 weeks following the completion of treatment [32,33]. This study was not able to demonstrate statistical benefit in corneal sensitivity and visual acuity.

Many surgical options are reserved for severe disease states. Amniotic membrane transplantation (AMT) is a simple surgical technique that can provide robust temporary protection to the cornea. A randomized control study showed similar times to complete re-epithelialization between AMT compared to more traditional approaches, such as tarsorrhaphy or bandage contact lenses [2,34]. Additionally AMT has been compared with autologous serum finding that both are similarly effective at treating corneal ulcers caused by NK [35]. The multilayer AMT, however, is more effective than autologous serum at healing deep corneal ulcers caused by herpetic NK [35].

A conjunctival (Gundersen) flap involves transferring a thin conjunctival covering over the cornea [36]. This is often employed as a final effort to protect the eye and provide anatomic integrity in severely thinned corneas approaching perforation [2]. A conjunctival flap occludes useful vision in the eye but maybe reversed [6,30]. This technique has several limitations including iatrogenic limbal cell deficiency, impaired intraocular pressure measurements, and potential future glaucoma surgery, though it can be particularly helpful as a temporizing measure when patient compliance with topical drops is in question [27].

Perforations due to NK may be difficult to manage. Cyanoacrylate gluing followed by application of a soft bandage contact or amniotic membrane is effective for treating small perforations. Corneal transplantation may be utilized for large perforations. Unfortunately, corneal transplantation rates are generally low because the cornea lacks trophic support and transplants are likely to develop ulcers [6].

Corneal neurotization is a recently established technique for treating severe NK with exciting potential to cure innervation deficits. It is more invasive and significantly less routine than other surgical therapies, requiring a motivated patient with disease progression despite more germane therapies. Reinnervation may be performed using direct nerve transfer of the ipsilateral infraorbital, supraorbital, or supratrochlear nerves or more commonly by nerve grafting. Nerve grafting employs the above-mentioned nerves or may use the contralateral supratrochlear or supraorbital branches in patients with complete ipsilateral V1 and V2 deficits [37]. Confocal microscopy has been utilized to confirm that reinnervation occurs ∼6 months after corneal neurotization [38].

Corneal neurotization was first performed using autologous nerve grafts [37], however, nerve allografts are now becoming increasingly reported as a safe, effective, and efficient approach [39,40]. A multicenter case series of 17 patients treated with processed nerve allografts found nearly ubiquitous corneal sensation recovery and decreased need for topical therapies. Neurotization may take several months for corneal sensation to return [39].

## Novel therapies

Matrix regeneration therapy is an emerging treatment for NK. ReGeneraTing Agent OTR4120 (RGTA) is a dextran derivative that functions as a heparan sulphate analog. It provides binding sites for growth factors and defense against proteolytic substances [41,42]. While numerous uncontrolled studies show favourable results including high rates of corneal healing [43,44] and decrease in ulcer area [45], some studies have less positive results [46]. Most studies disclosed no or very minimal adverse effects. Comparative and randomized studies of matrix regeneration therapy are needed.

Plasma rich in growth factors (PRGF) is made of autologous platelet protein extracts which contain numerous growth factors. It has previously been used in maxillofacial, reconstructive, and orthopaedic surgery to heal skin, mucous membranes, and subcutaneous tissue [47]. Sabater et al. showed that PRGF can successfully treat ocular surface disease, including neurotrophic keratopathy [48]. Lopez-Plandolit et al. quantified the levels of the following growth factors found in undiluted PRGF: platelet-derived growth factor, epithelial growth factor, vascular endothelial growth factor, hepatocyte growth factor, and fibroblast growth factor [47]. Rao et al. studied autologous plasma in 11 eyes of six patients reporting improvement in best-corrected visual acuity, decrease in fluorescein staining, increased corneal sensitivity, and increased number, length, width, and density of subepithelial nerves after autologous plasma treatments [49].

Thymosin β4 is a naturally occurring amino acid with wound healing, anti-inflammatory, and synaptogenic properties. It has previously been studied in animal models. Dunn et al. showed that Thymosin β4 resulted in a decrease in size of epithelial defects in a case series on four patients who were treated with Thymosin β4 for one month and then followed by a one-month observational period [50]. One patient, whose epithelial defect was stable before initiation of therapy, continued to have epithelial wound healing during the observational period, despite cessation of Thymosin β4 [50]. Further studies are needed to determine the optimal dose or length of treatment. Thymosin β4 is currently being studied in a clinical trial for patients with dry eyes (NCT01387347). Little data has been published on the use of TB4 for NK since Dunn’s small case series from 11 years ago.

Finally, nicergoline is an ergoline derivative that is used to treat cognitive impairment after dementia and stroke. Oral administration has been shown to increase NGF secretions in rat tears and lacrimal glands and lead to an increased rate of rat corneal healing [51]. A prospective non-comparative study of 27 eyes reported an 85% rate of complete healing of epithelial defects, and improved best-corrected visual acuity, increased corneal sensitivity, and tear nerve growth factor levels [52]. Comparative and randomized nicergoline formulations are needed.

## Conclusion

Neurotrophic keratopathy is a disease associated with significant morbidity, often challenging for patients and clinicians alike. Early evaluation, staging, and appropriate treatment are vital to maintaining corneal integrity and preventing disease progression. Numerous topical treatment modalities may be employed to stabilize the disease. Cure of NK with medical and surgical re-innervation has been breakthrough therapies for the appropriate candidates. Novel therapies on the horizon may further the provider’s armamentarium in treating patients at various stages of involvement. Further research is needed to continue to expand our knowledge on disease prevention, identification, and treatment.

## Data Availability

Data sharing is not applicable to this article as no new data were created or analyzed in this study.
